# Short-term imaging response after drug-eluting embolic trans-arterial chemoembolization delivered with the Surefire Infusion System^®^ for the treatment of hepatocellular carcinoma

**DOI:** 10.1371/journal.pone.0183861

**Published:** 2017-09-01

**Authors:** Alexander Y. Kim, Shelby Frantz, Pranay Krishnan, Danielle DeMulder, Theresa Caridi, George Emmett Lynskey, James B. Spies

**Affiliations:** Department of Radiology, Division of Interventional Radiology, Medstar Georgetown University Hospital, NW, Washington, DC, United States of America; Chang Gung Memorial Hospital Kaohsiung Branch, TAIWAN

## Abstract

**Purpose:**

To review the initial imaging responses after drug-eluting embolic trans-arterial chemoembolization (DEE-TACE) delivered with the Surefire Infusion System ^®^ for the treatment of hepatocellular carcinoma (HCC).

**Methods:**

Single center retrospective evaluation of patients who underwent DEE-TACE for HCC, delivered with SIS. Information was gathered from available medical records. Treatment response rates were assessed using the modified Response Evaluation Criteria in Solid Tumors criteria. Assessment of adverse events was categorized per Common Terminology Criteria for Adverse Events version 4.03.

**Results:**

Twenty-two patients with 39 hepatocellular carcinoma lesions were treated with the surefire infusion system. Complete response was demonstrated in 32% of patients and 54% of lesions after a single treatment session. Overall disease response was demonstrated in 91% of patients and 85% of lesions after a single treatment. No grade 3 or higher elevations in liver function tests were demonstrated in the short-term.

**Conclusion:**

SIS delivered DEE-TACE leads to a higher than expected initial response in patients with HCC.

## Introduction

Trans-arterial chemoembolization (TACE) is the standard of care for patients with intermediate stage hepatocellular carcinoma (HCC)[1]. This recommendation is largely based on two randomized controlled trial and a systemic review demonstrating a survival advantage of TACE over best medical management for HCC[2,3]. TACE is also often utilized as a strategy to maintain or downstage patients within United Network for Organ Sharing (UNOS) transplant criteria[4–6].

Despite improved patient outcomes with TACE, tumor response rates in published studies are modest. Based on modified Response Evaluation Criteria in Solid Tumors Criteria (mRECIST) or European Association for the Study of Liver (EASL) criteria, various prospective evaluations have demonstrated complete and partial response rates of ~25% and ~25–50% of patients, respectively, after multiple TACE sessions[7,8]. Outcomes after a single TACE treatment are poor with complete response rates of <10%[7]. Improving response rates to therapy may lead to a survival advantage as response to TACE, especially the response after initial treatment, has been linked to overall survival[9,10].

Much of the research performed to improve outcomes after TACE has focused on the two components of TACE: the chemotherapy drug and the embolic material[11,12]. Various types and doses of chemotherapy agents including Doxorubicin, Cisplatin, and others have been assessed for their efficacy when used as the mainstay chemotherapy drug in TACE[13–15]. Evaluations of different embolic material in varying sizes have been studied in attempts to achieve greater response rates[16,17]. Studies have been performed to determine if the combined delivery of chemotherapy and embolic particles through drug-eluting beads (DEB-TACE) is equivalent or superior to the conventional method cTACE[11,18,19], wherein chemotherapy is delivered prior to the embolic material. The method of delivering these drugs and particles, however, has not been an area of robust research interest despite its potential to impact patient outcomes.

The Surefire Infusion System (SIS) is an anti-reflux microcatheter which has a funnel-shaped, self-expanding tip (Surefire Infusion System; Surefire Medical, Inc., Westminster, CO). The tip partially collapses during systolic flow but expands during diastole, providing a barrier to prevent particle reflux. Although initially designed with reflux protection in mind, studies in animal models have demonstrated enhanced embolization efficiency with SIS, with deeper particle penetration when compared with a standard microcatheter (SM)[20]. Early human studies also suggest a role in augmenting tumor uptake of particles infused with SIS[21].

We theorized that the enhanced embolization efficiency,deeper particle penetration and enhanced tumor uptake demonstrated in earlier studies may lead to improved outcomes after TACE. The aim of this study was to evaluate the initial treatment response and adverse events after SIS-TACE.

## Methods

Institutional review board approval was obtained from the Medstar Georgetown University IRB to perform this retrospective evaluation. Consent requirement was waived for this retrospective evaluation. The electronic medical record system was searched for patients undergoing TACE for HCC between January 2015 and June 2016 utilizing SIS as the treatment microcatheter. Patients were excluded from evaluation if they were treated with both a SM and SIS prior to follow up imaging. Patients undergoing other HCC directed treatments such as RFA were also excluded from evaluation.

All patients were evaluated at the interventional radiology clinic by one of the study authors. Available cross sectional imaging, functional status and laboratory values were evaluated to assess for treatment eligibility. All patients had either biopsy proven or cross sectional imaging confirmation of HCC. Patients considered for treatment with SIS met standard criteria to undergo TACE including: Barcelona clinic liver cancer (BCLC) stage A or B, no evidence of macrovascular invasion on imaging, and total bilirubin level less than 3.0 mg/dL. Detailed baseline characteristics of treated patients are demonstrated in Table 1.

**Table 1:** Baseline patient characteristics.

Age		59.5 (range 36–74)
Sex		
	Male	17
	Female	5
Etiology		
	HBV	4
	HCV	12
	Alcohol	4
	NASH	2
Child-Pugh Score		
	A	16
	B	5
	C	1
BCLC Stage		
	A	10
	B	12
Tumor Distribution		
	Unilobar	17
	Bilobar	5
Number of Lesions		39 (range 1–6)
Average Tumor Diameter		2.6 cm (range 0.6–7.7cm)
Prior TACE		12
Total Bilirubin		0.9 mg/dL (range 0.2–2.5 mg/dL)
Alkaline Phosphatase		120 u/L (range 64–217 u/L)

**Fig 1:**
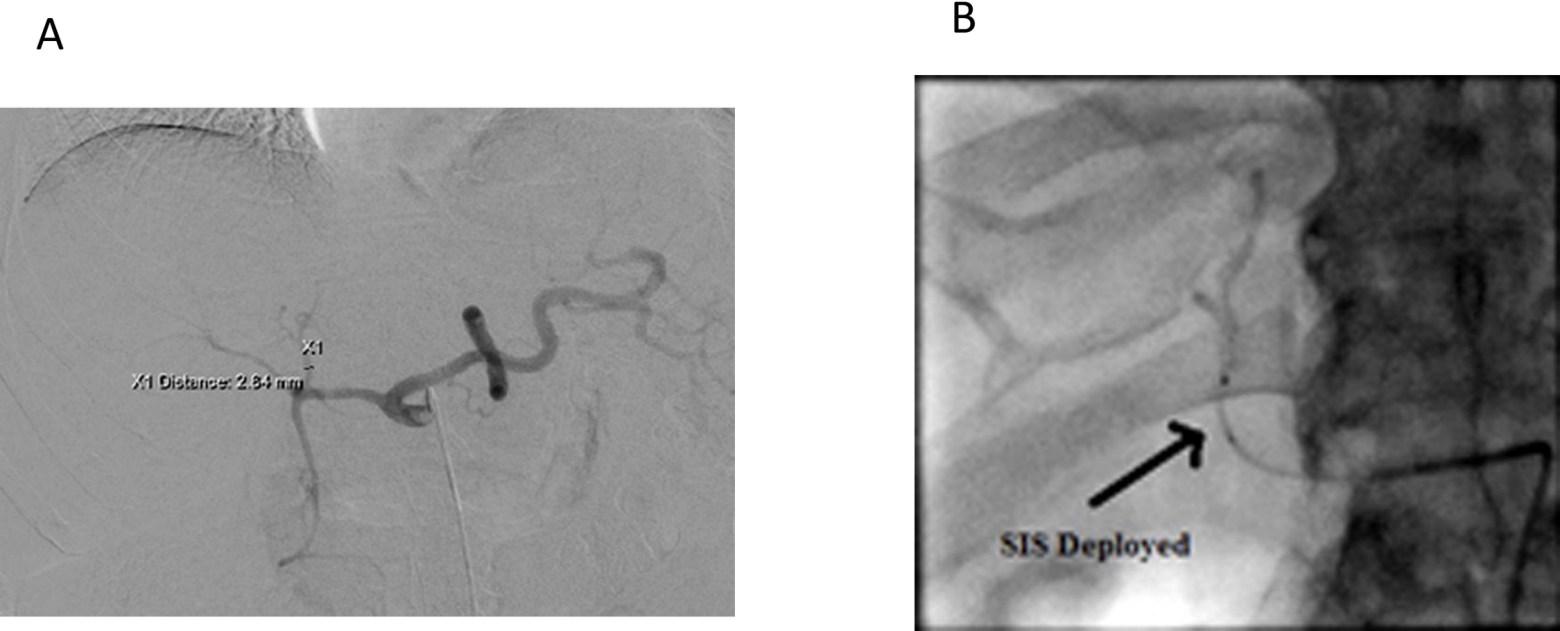
**(A)** Measurement of a target vessel. **(B)** Infusion of DEE-TACE through an expanded SIS.

All procedures were performed under conscious sedation. A guide catheter (Surefire Medical, Westminster, Co) was advanced through a sheath placed in the right common femoral artery and used to perform superior mesenteric and celiac angiograms. CBCT was used at the discretion of the operating physician to identify tumor feeding vessels. The vessel supplying the intended treatment region was identified and the diameter of the vessel was measured using the angiographic system software (Siemens, Munich, Germany). The appropriately sized SIS was selected based on the diameter of the vessel where treatment was planned (Fig 1). The SIS was prepared as per manufacturer recommendations. The SIS was advanced over a 0.016” Fathom wire (Boston Scientific, Marlborough, MA). A final angiogram was performed prior to delivery of particles to confirm tumor supply. Each treatment was performed with a single vial of 100 micron Oncozene (Boston Scientific, Marlborough, MA) or 70–150 micron LC-beads (BTG, London, UK) loaded with 50mg of Doxorubicin. Further bland embolization with 100–300 or 300–500 micron Embospheres (Merit Medical, South Jordan, Utah) were performed at the operating physicians’ discretion to achieve complete stasis.

All post-embolization digital subtraction angiography (DSA) were performed with the microcatheter tip retracted. Stasis was defined as no forward flow of contrast beyond a second order branch from where the microcatheter tip, angiographic evidence of reflux during embolotherapy despite tip expansion, or visualization of intra-hepatic collaterals during embolization.

Follow-up MRI was obtained 4–8 weeks after treatment. Tumor response assessment was made according to mRECIST criteria by two independent fellowship trained body radiologists with 6 and 2 years of experience. Inter-observer variability was resolved by a third radiologist with 5 years of experience. For purposes of this evaluation patients undergoing planned staged treatments targeting lesions in different vascular territories (i.e. right and left hepatic artery treatments) were considered to have undergone a single treatment session after completion of all treatments. Laboratory assessments were made prior to initial treatment with SIS and within 1 week of follow up imaging. Adverse events were categorized per Common Terminology Criteria for Adverse Events (CTCAE) version 4.03, based on available electronic medical records.

## Results

Thirty-three patients were treated with SIS during this time period. Four patients were excluded due having been treated with both SIS and SM prior to follow up imaging. Two patients underwent radiofrequency ablation and liver transplantation, respectively, prior to follow up imaging. Five patients had no follow up studies at time of evaluation.

A total of 22 patients with 39 separate HCC lesions underwent 28 treatments with SIS. Ten patients (45%) underwent de novo treatment with SIS whereas 12 patients (55%) were previously treated with a SM. Ten SIS treatments (36%) were delivered in a lobar fashion and 17 (64%) were from a segmental/subsegmental branch.

**Fig 2:**
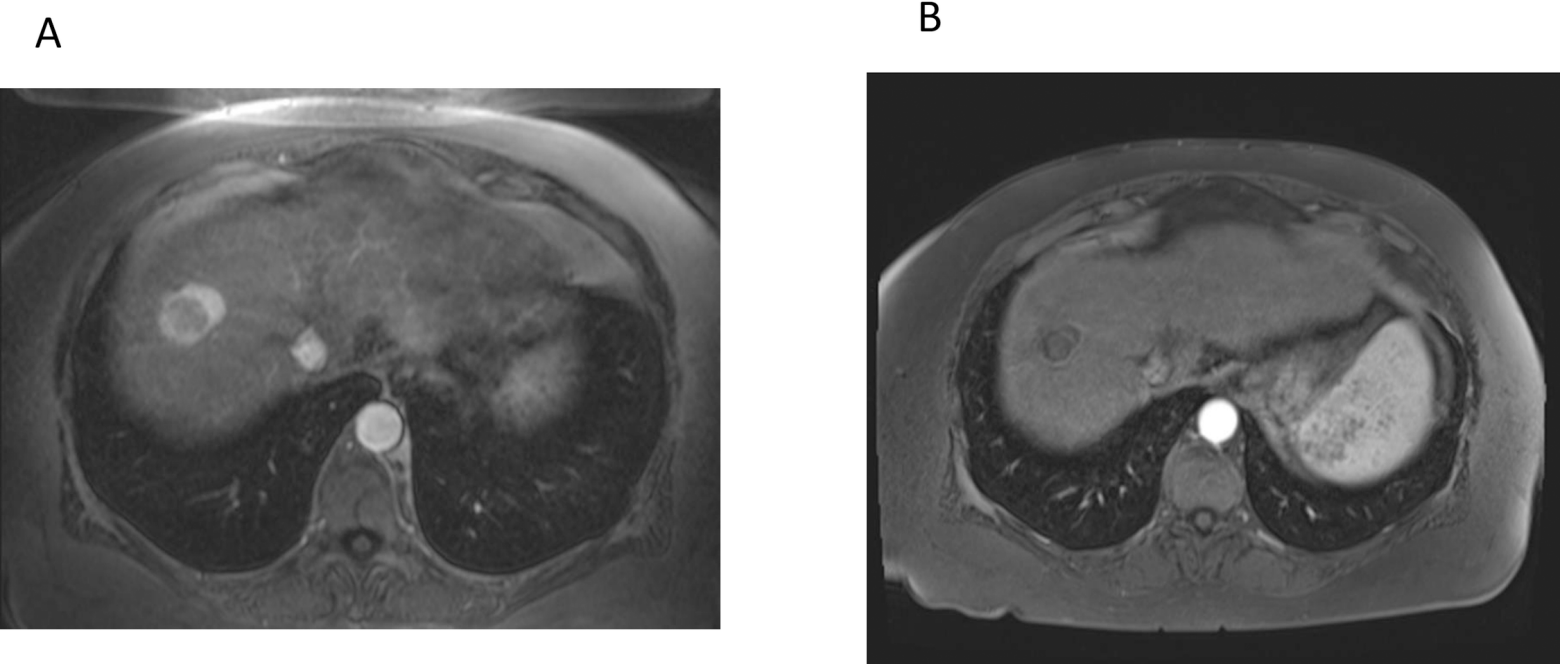
**(A)** Initial MRI demonstrating a 3.5 cm hyperenhancing right lobe mass, LIRADS-5 for HCC. **(B)** Post embolization MRI demonstrating complete response to treatment.

**Fig 3:**
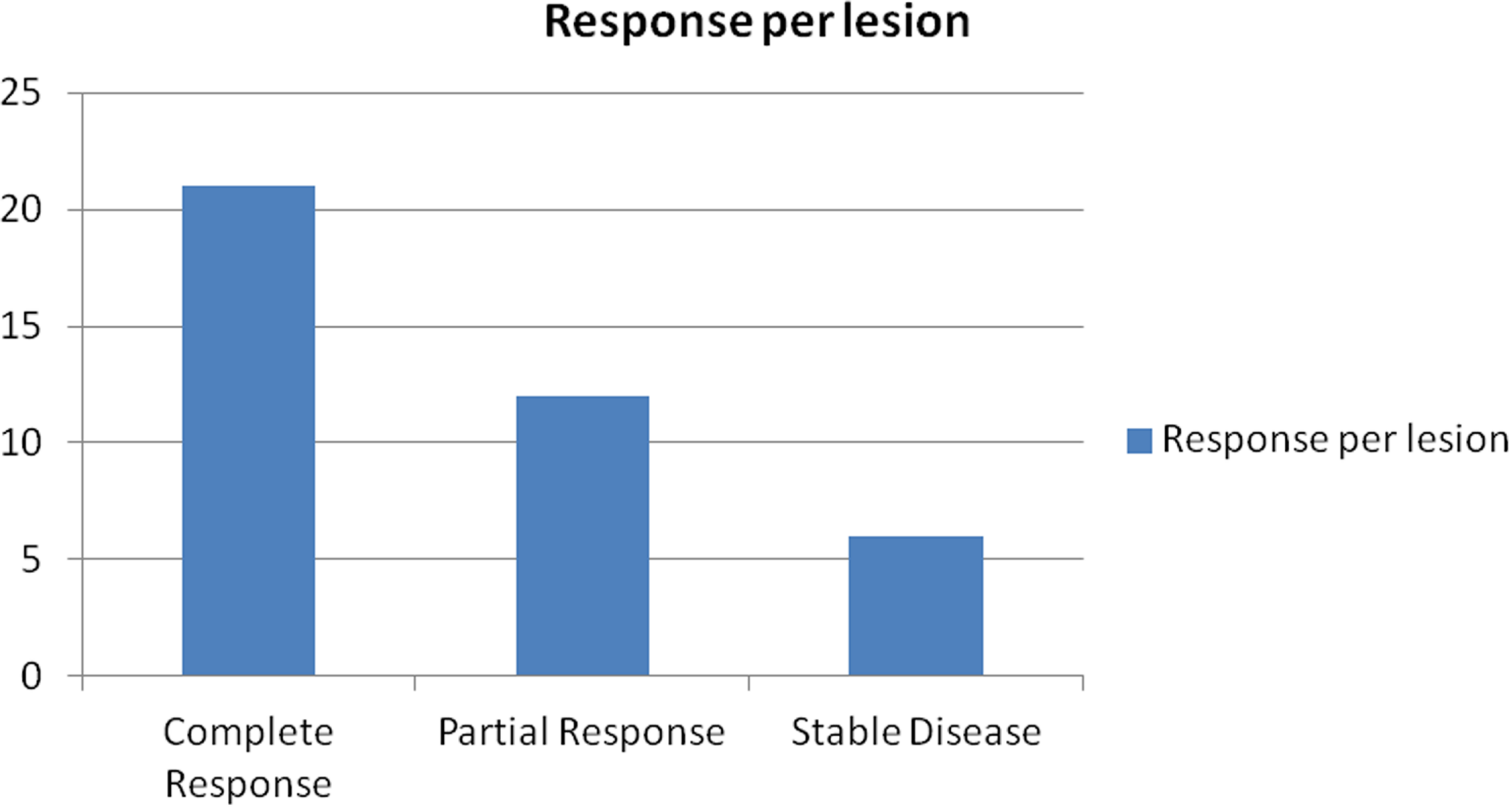
Disease response per lesion based on mRECIST criteria.

Seven patients (32%) with 10 lesions demonstrated a complete response (CR) to treatment on their short-term follow up (Fig 2). Fourteen patients (61%) with 27 lesions demonstrated partial response (PR) to treatment. Lesion specific outcomes were CR in 21 of 39 lesions (54%), PR in 12/39 (31%) and stable disease (SD) in 6/39 (15%). The overall disease response rate and disease control rates were 85% (33/39) and 100% (39/39), respectively. (Fig 3).

Twelve patients had disease burden outside of UNOS transplant criteria at the time of SIS treatment. Eleven patients (92%) had their disease successfully down-staged to be within UNOS transplant criteria after their initial SIS treatment.

No grade 3 or higher elevation of alkaline phosphatase or total bilirubin was seen at follow up. The mean total bilirubin and alkaline phosphatase at baseline were 0.9 mg/dL (range 0.2–2.5 mg/dL) and 120 u/L (range 64–217 u/L), respectively. The mean total bilirubin and alkaline phosphatase at follow up were 0.8 mg/dL (range 0.2–1.9 mg/dL) and 128 u/L (range 53–226 u/L), respectively.

## Discussion

Patient survival after TACE is thought to be directly correlated to disease response to treatment, based on imaging mRECIST and EASL criteria Patients demonstrating imaging response after TACE have superior survival to those with stable disease or disease progression[10]. Furthermore, patients demonstrating complete response to therapy have superior outcomes compared to those with partial response. For patients achieving complete response to TACE after a single treatment session, the overall median survival may approach that of surgical resection and/or transplantation[9].

This survival advantage appears to be true even for those undergoing resection or transplantation following TACE. In their evaluation of 373 consecutive patients, Allard et al. demonstrated an overall median survival and progression free survival advantage in resection and transplant patients achieving complete pathologic response after pre-surgical TACE[22].

Current literature demonstrates an overall response rate of ~50–70% in HCC patients treated with multiple sessions of TACE[8,23,24]. Malagari et al demonstrated a complete response rate of 8% after initial TACE, with an improvement to 23% after multiple sessions[18]. This is in contrast with our finding of complete and overall response rates of 30% and 91% of patients after a single treatment session TACE with SIS.

Our results are especially impressive given the baseline demographics of the treatment group. Thirteen of the 23 patients had prior treatment with SM with residual or progressive disease. Twelve patients had tumor burden outside of UNOS transplant criteria at time of initial SIS TACE. Furthermore, 36% of the treatments were delivered in a lobar fashion–due to tumor burden–which is thought to lead to poorer outcomes compared with subselective TACE. Despite this, we were able to achieve complete response in 53% of targeted lesions after a single treatment session.

We theorize that one explanation for the apparent improved outcomes demonstrated in our study may be due to the improved tumor targeting when delivering particles using SIS. This is supported by the work of Pasciak et al. who demonstrated enhanced tumor uptake of technetium labelled micro-aggreggated albumin (Tc99-MAA) when delivered through SIS compared with a standard microcatheter[21].

Another potential mechanism to explain our outcomes is the potential to achieve a higher degree of embolic effect with deeper particle penetration with SIS as demonstrated by Arepally et al. in their pig model comparison of the SIS versus a standard 4 Fr catheter[20].

A third potential mechanism to explain our findings is supported by the work of Irie et al.[25] who demonstrated that the use of a balloon occlusion catheter during conventional TACE (cTACE) led to enhanced lipiodol deposition in tumor in 39/43 treated lesions. They found that infusion of cTACE with a balloon occlusion catheter lead to an initial even distribution of lipiodol in a segmental territory followed by preferential uptake of lipiodol in tumor in 39 or 43 treatments. They suspect that development of intrahepatic collateral supply supplying tumor may lead to the persistent tumor uptake of particles due to the lower pressure downstream territory.

Although the practice pattern varies widely, for many interventionalists a standard end point for TACE is when contrast/particle reflux is visualized during embolization[26]. We suspect that even at the point of reflux during embolization with a SM, complete stasis of blood flow is often not reached. This may be one cause of suboptimal response rates which are established in various prospective trials of TACE.

Despite the higher degree of embolic effect achieved with SIS, we found no evidence of significant treatment related hepatotoxicity. Also, despite the presumed increased level of embolization, there was no evidence of biloma formation in any follow up studies.

One difficulty in performing SIS TACE is evaluating the optimal end point of treatment. The goal for our treatments was to achieve complete stasis while being careful not to over-embolize to minimize potential risks of hepatotoxicity or bile duct injury. Our solution was to routinely retract the tip and perform angiograms for assessment of forward flow at defined intervals of every one-half vial of infused embolic after infusion of 2 vials. We also stopped the embolotherapy when contrast reflux was seen despite the catheter tip being expanded or identification of intra-hepatic collaterals forming during embolization. However a more objective end-point measure is needed. One potential theory currently being investigated is to correlate treatment end-points with the hepatic to systemic arterial pressure gradient[27].

We believe our preliminary findings are promising and suggest that DEE-TACE delivered with SIS may provide therapeutic benefit for patients with HCC. There are, however, inherent limitations to our study due to its retrospective nature. Despite the improved response rates demonstrated in our series, a direct survival benefit cannot be inferred. We did not directly compare our study population with a control group treated with an end hole catheter. In addition, given the additional cost of SIS versus SM, cost-benefit analyses are needed. Additional studies are needed for confirmation of our findings. Prospective evaluations of SIS for DEE-TACE are currently ongoing to answer some of these questions.

## Supporting information

S1 File Deidentified data. Deidentified imaging response and laboratory data used in study.
(XLSX)
